# m1A Regulatory gene signatures are associated with certain immune cell compositions of the tumor microenvironment and predict survival in kidney renal clear cell carcinoma

**DOI:** 10.1186/s40001-023-01292-3

**Published:** 2023-09-07

**Authors:** Linjun Zhou, Weidong Zhou, Yuan Li, Ruifang Hua

**Affiliations:** 1 Department of Nephrology, Ganzhou Hospital of Guangdong Provincial People’s Hospital, Ganzhou Municipal Hospital, Ganzhou, China; 2 Department of Emergency, Zhanggongqu Hospital Of Traditional Chinese Medicine, Ganzhou, China; 3 Department of Clinical Laboratory, Yangpu Hospital, Tongji University School of Medicine, Shanghai, China; 4 Department of Molecular Diagnostics and Experimental Therapeutics, Beckman Research Institute of City of Hope, Biomedical Research Center, Monrovia, CA 91016 USA

## Abstract

Adenosine N1 methylation (m1A) of RNA, a type of post-transcriptional modification, has been shown to play a significant role in the progression of cancer. The objective of the current research was to analyze the genetic alteration and prognostic significance of m1A regulators in kidney renal clear cell carcinoma (KIRC). Genomic and clinicopathological characteristics were obtained from 558 KIRC patients in the Cancer Genome Atlas (TCGA) and Gene Omnibus Expression (GEO) databases. Alterations in the gene expression of ten m1A-regulators were analyzed and survival analysis was performed using the Cox regression method. We also identified three clusters of patients based on their distinct m1A alteration patterns, using integrated analysis of the ten m1A-related regulators, which were significantly related to overall survival (OS), disease-free survival (DFS) and tumor microenvironment (TME) immune cell infiltration cells in KIRC. Our findings showed that m1A alteration patterns have critical roles in determining TME complexity and its immune cell composition. Furthermore, different m1A expression patterns were significantly associated with DFS and OS rates in KIRC patients. In conclusion, the identified m1A RNA modification patterns offer a potentially effective way to classify KIRC patients based on their TME immune cell infiltration, enabling the development of more personalized and successful treatment strategies for these patients.

**Supplementary Information:**

The online version contains supplementary material available at 10.1186/s40001-023-01292-3.

## Introduction

Kidney cancer is one of the most prevalent types of cancer in both males and females. The number of patients with kidney cancer has increased in the past two decades, comprising up to 2–3% of all new occurrences of cancer [1]. Renal cell carcinoma (RCC), is the most frequent type of kidney cancer, accounting for up to 85% of all cases [2]. RCC affects approximately 400000 individuals annually worldwide [3]. It mostly affects males over the age of 60 [4]. Among different pathological subtypes of RCC, kidney renal clear cell carcinoma (KIRC) is the most common subtype, comprising 75% of all renal cell cancer cases [5]. Surgical resection is often the only effective treatment option for KIRC, since it is generally resistant to chemotherapy and radiotherapy [6]. However, even with early surgical intervention, 30% of patients with localized tumors will subsequently show metastasis [7]. Therefore, early identification of KIRC patients with high metastasis risk could be useful for a more accurate prediction of clinical outcome. Furthermore, effective tumor immunotherapy biomarkers will be advantageous to improve the response rate. In addition, determining patient subgroups that could benefit from certain targeted therapies requires urgent investigation of the factors involved in the carcinogenesis and progression of KIRC.

Moreover, omics approaches, encompassing genomics, transcriptomics, proteomics, and metabolomics, have revolutionized the management of different cancer types as well as RCC [8]. Early detection and diagnosis benefit from omics-derived biomarkers, enabling timely intervention for improved outcomes [9]. Fujiwara et al.'s study underscores the significance of omics studies in cancer management, with a focus on early detection and diagnosis through omics-derived biomarkers. They highlighted that various biomolecules, including germline DNA polymorphisms, transcriptomic dysregulations, circulating molecules, and gut microbiota, contribute to predicting hepatocellular carcinoma (HCC) risk, enhancing the potential for accurate early diagnosis and timely intervention [10]. Furthermore, the study by Qu et al. highlights the crucial role of omics studies in managing ccRCC. Through a comprehensive proteogenomic analysis, the study reveals metabolic dysregulation, an amplified immune response, and molecular subtypes in ccRCC [11]. The identification of potential markers and drug targets emphasizes how omics approaches provide insights into disease complexity, enabling personalized treatment strategies and improved patient outcomes. In sum, omics transforms RCC management, driving us toward personalized therapeutic approaches, early interventions, and improved patient outcomes [12].

Major cellular functions, such as cell differentiation, critical cellular signaling pathways, and cell metabolism are partly regulated at the post-transcriptional level through biochemical modifications of RNA [13]. To date, more than 170 post-transcriptional biochemical modifications of RNA have been reported in noncoding RNAs and mRNAs, generating functional differences [14]. The most common types of such alterations are N1-methyladenosine (m1A) [15], N6-methyladenosine (m6A) [16], pseudouridine (*Ψ*) [17], and 5-methylcytosine (m5C) [18] modifications. m1A modification is a type of dynamic reversible methylation at the N1 position of adenosine in mammalian cells, contributing to RNA secondary structure stabilization and alteration in protein − RNA binding interactions [19]. Methyltransferases, binding proteins, and demethylases, the so called “writers” (TRMT6/61A, TRMT61B, and TRMT10C), “readers” (YTHDC1, YTHDF1, YTHDF2, and YTHDF3), and “erasers” (FTO, ALKBH1, and ALKBH3), respectively, are the major regulators of m1A modification [19]. In summary, “writers” and “erasers” methylate and demethylate, respectively, while “readers” identify and bind to methylation spots [20]. The function of each "reader" determines the endpoint of the modified RNA including splicing, stability and/or translation [21]. The current studies suggest that m1A regulators' genetic mutation may affect the transcription and translation processes, leading to abnormal cell proliferation and tumorigenesis [22]. In this case, according to Shi et al., m1A-related regulatory genes are essential for the tumorigenesis of hepatocellular carcinoma and have prognostic and diagnostic value [23]. Zheng et al. reported that dysregulation of the m1A-related regulatory genes can be identified as prognostic biomarkers for pancreatic cancer [24]. Moreover, Gao et al. revealed that different RNA modification patterns are correlated with tumor immune microenvironment characteristics in oral squamous cell carcinoma. Based on their results, two different clusters on the basis of m1A gene signature were identified, which were correlated with prognosis and immune microenvironment features [25]. With regard to KIRC, YTHDF2, a well-known m6a reader protein, has been shown to be an indicator of higher immune cell infiltration and its higher expression was associated with longer overall survival rate [26]. Nonetheless, the role of m1A modification is not completely understood in KIRC and a broader investigation of these regulators would aid in elucidating the possible functions of m1A modifications in different physiological and pathological processes.

In the present study, we analyzed the clinicopathological data from a cohort of 558 KIRC patients from The Cancer Genome Atlas (TCGA-KIRC) and Gene Expression Omnibus (GEO) databases. We then analyzed the alterations in the profiles of ten m1A-related regulatory genes and identified the associations between their modification patterns, expression data, somatic mutations, and clinicopathological characteristics including prognosis in KIRC patients. In addition, different m1A modification patterns were identified and their correlation with immune infiltrating cells within the tumor microenvironment (TME) was investigated. To further investigate the functions of the identified differentially expressed genes (DEGs), gene set enrichment analysis (GSEA) was carried out. To discover the putative signaling pathways Kyoto Encyclopedia of Genes (KEGG) and Genomes analyses were performed. Ultimately, we evaluated the association between the expression patterns of m1A regulators and disease-free survival (DFS) in KIRC patients.

## Methods

### Data acquisition

The Gene Expression Omnibus (GEO) and The Cancer Genome Atlas (TCGA) databases were used to download clinical information as well as gene expression and RNA-sequencing data of KIRC patients. In summary, two eligible KIRC cohorts (TCGA-KIRC and GSE22541) were identified suitable for our analysis. The data of the TCGA-KIRC dataset was downloaded through the UCSC Xena portal (https://xenabrowser.net/hub/). Somatic mutation data was further obtained from the TCGA-KIRC cohort for further analysis. “GEOquery” package of R was utilized to obtain the expression profiles of GSE22541 patients. The final expression values were obtained after quantile normalization and log2(*x* + 1) transformation. When a single gene had numerous probes, the mean expression was calculated. Genes with no expression values were excluded from the study. Patients without prognostic data were not included in the analysis. Table 1 provides an overview of the clinical characteristics of the cohorts included in the present study.

**Table 1:** Clinical characteristics of the cohorts included in this study

	TCGA-KIRC (*n* = 534)	GSE22541 (*n* = 24)
Sex		
Male	348 (64.1%)	13 (54.2%)
Female	186 (35.9%)	11 (45.8%)
Age		
Median	61	–
Range	26–90	–
TNM Stage		
I	268 (50.2%)	10 (41.7%)
II	58 (10.9%)	9 (37.5%)
III	123 (23.0%)	5 (20.8%)
IV	82 (15.4%)	0 (0%)
Not available	3 (0.5%)	–
Grade		
G1	14	0
G2	231	18
G3	206	6
G4	75	0
Gx	8	0
Follow up		
Alive	361 (67.6%)	–
Death	173 (32.4%)	–
Median OS	38.8	72.5

### DEG identification, somatic mutation analysis, and prognostic study of the 10 m1A regulatory genes

To identify survival-related regulatory genes among the 10 m1A regulators, a univariate cox regression analysis was conducted. Forest plot and Kaplan–Meier curves were depicted to identify the prognostic significance of the studied RNA regulators. The Spearman correlation method was used to analyze the relationship between the 10 m1A-related regulators. In order to obtain differentially expressed m1A-related regulatory genes (m1A-related DEGs) between the tumor and normal samples or between different subtypes, the “limma” package of *R* was used and the adjusted p value cutoff was set as < 0.05. Ultimately, the Maftools *R* package was used to investigate the tumor mutational burden related to the 10 m1A regulatory genes.

### Unsupervised hierarchical clustering of 10 m1A-related methylation regulators in KIRC

The 10 m1A-related regulators including ALKBH1, ALKBH3, TRMT10C, TRMT6, TRMT61A, TRMT61B, YTHDC1, YTHDF1, YTHDF2, and YTHDF3 were selected to construct different modification patterns. Subsequently, unsupervised hierarchical clustering analysis was performed using the *R* package. Accordingly, 558 patients were classified into three distinct subgroups (“C1”, “C2”, and “C3”). The “pheatmap” package of *R* was implemented to visualize the heatmap of these three clusters.

### Immune and stromal cell infiltration analysis

The ESTIMATE algorithm was applied to evaluate the degrees of immune cell infiltration among different clusters. The immune, stromal, and ESTIMATE scores were calculated and then a gene set of human immune cell subtypes was retrieved from published references. The single-sample gene set enrichment analysis (ssGSEA) was performed to evaluate the relative abundance and levels of activity of each immune cell type in the TME of KIRC. Moreover, to evaluate the differences in the immune subtypes, the proportion of infiltrating immune cells in KIRC patients with their expression patterns were assessed using CIBERSORT.

### Functional enrichment analysis

Gene ontology (GO) and KEGG pathway analysis were conducted using the clusterProfiler and ggplot2 *R* packages. The inclusion criteria were set as *p* < 0.01 and *q* < 0.05.

### Construction and validation of m1A gene signature

A random forest classifier was constructed by random based m1A gene’s expression in TCGA-KIRC dataset. Using the same classifier, subtype labels of patients from independent cohorts were classified. Before classifier construction and validation, the expression values of all the cohorts were Z score normalized, respectively.

## Results

### The landscape of m1A-related regulatory genes in KIRC

**Fig. 1:**
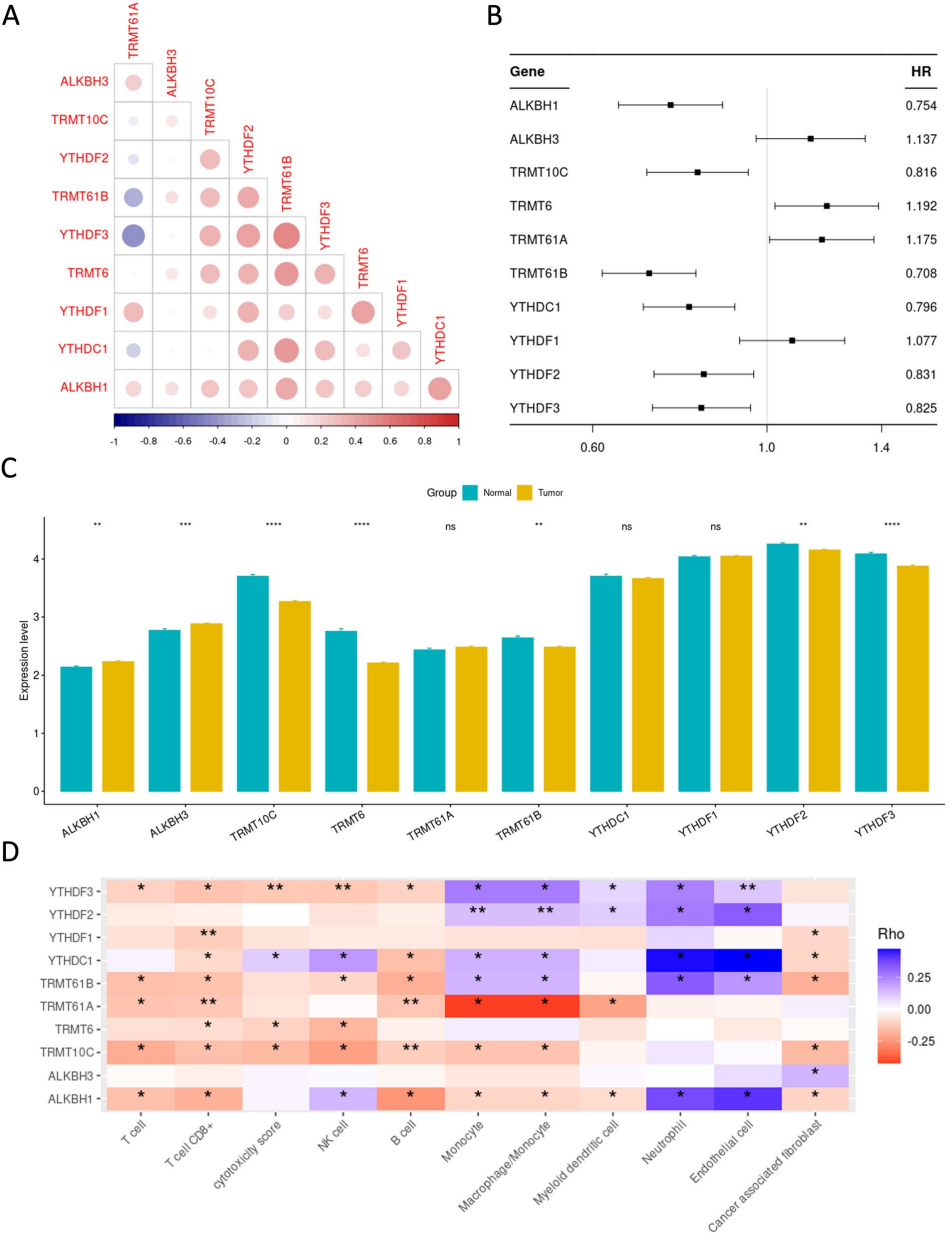
The landscape of m1A-related regulators in KIRC. **A** Crosstalk and relationship of the ten m1A regulators in the TCGA-KIRC patients. **B** Univariate cox analysis of the ten m1A-related regulatory genes **C** Differentially expressed genes of the ten m1A-related regulators between tumor and normal samples of the TCGA-KIRC cohort. **D** Correlation of the ten m1A-related regulators with tumor infiltration of immune cells in the TCGA-KIRC patients

Briefly, 10 m1A regulatory genes were analyzed in the present study, including four writers (TRMT61A, TRMT61B, TRMT6, and TRMT10C), four erasers (YTHDF1, YTHDF2, YTHDF3, and YTHDC1), and two readers (ALKBH1 and ALKBH3). Initially, the relationship between the selected m1A regulatory genes were studied in the TCGA-KIRC dataset. As shown in Fig. 1A, TRMT61B-YTHDF3 and TRMT61A-YTHDF3 had the strongest and weakest relationships among others, respectively. In addition, a comparison of the expression data revealed that 7 out of the 10 m1A regulators were dysregulated in the TCGA-KIRC patients, while the remaining three (TRMT61A, YTHDC1, and YTHDF1) did not show significant changes. Furthermore, clinical data from the TCGA-KIRC dataset was extracted and univariate Cox analysis on the 10 regulatory genes was implemented. The findings revealed that 2 out of the 10 genes (TRMT6 and TRMT61A) were strongly associated with poor prognosis of KIRC patients (Fig. 1B). Among the regulatory genes, ALKBH1 and ALKBH3, which are methylation erasers, were upregulated and TRMT10C, TRMT6, TRMT61B, YTHDF2, and YTHDF3 were downregulated in tumor samples in comparison to normal tissues (Fig. 1C). In addition, we identified a strong association between the ten m1A regulators and the present TME-infiltrating immune cells employing the Spearman's correlation analysis and GSEA method for infiltrated immune cells. Our results indicated that most of these regulators have varied degrees of association with immune cells. Meanwhile, the regulator YTHDC1 was positively associated with the infiltration of most of the immune cells (Fig. 1D). Based on these findings, it can be concluded that m1A regulators are among critical role players in the continuous dynamics of the immune microenvironment as KIRC progresses. Finally, mutation frequency of the selected m1A regulators were analyzed in TCGA-KIRC. Our findings revealed that 7 out of 558 KIRC samples carried m1A-related regulatory mutations, which ranged from 14 to 43% for 5 genes (YTHDC1, ALKBH1, TRMT61B, YTHDF2, and YTHDF3) and YTHDC1 was the top-ranked gene with 43% mutation among others (Additional file 1: Figure S1).

### Unsupervised hierarchical clustering based on the m1A modification patterns and its correlation with different tumor infiltrating cells

**Fig. 2:**
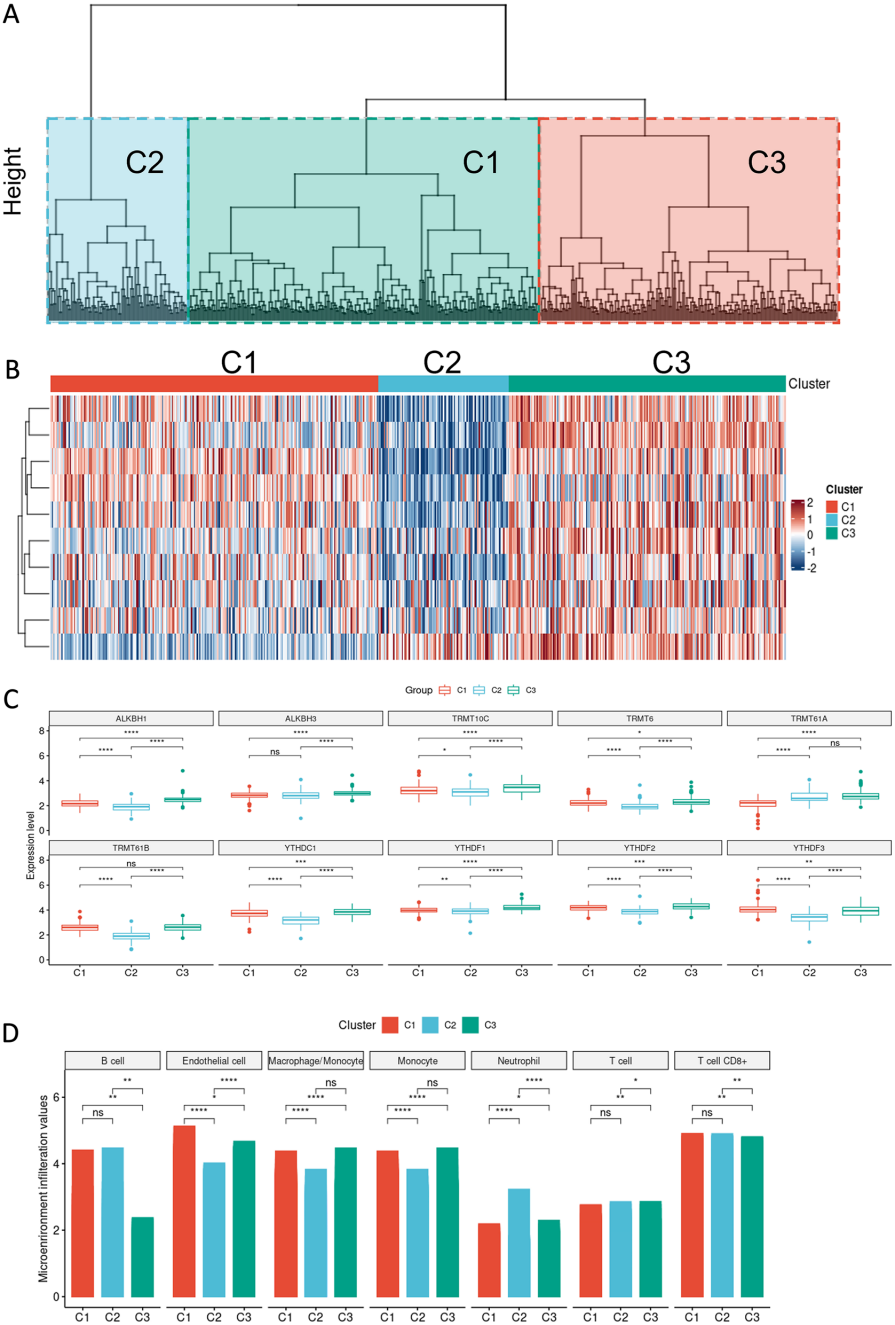
m1A modification patterns. **A** Dendrogram of hierarchical clustering of the ten m1A-related regulators in the TCGA-KIRC cohort. **B** Heatmap of the identified modification patterns in the TCGA-KIRC cohort. **C** Distribution of the ten m1A-related regulators in the three distinct modification patterns. **D** The proportion and abundance of the 7 tumor infiltrating cell types in the identified modification patterns

Five hundred and thirty-four KIRC patients from the TCGA dataset (the Cancer Genome Atlas-Kidney Renal Clear Cell Carcinoma) were included in unsupervised clusters for categorizing the various m1A modification patterns based on the expression levels of the ten m1A regulators. Finally, we identified three distinct m1A modification patterns, which were classified as ‘‘C1, C2, and C3’’. These clusters are depicted in a dendrogram in Fig. 2A as well as in a heatmap in Fig. 2B. Subsequently, the expression differences of the ten m1A-related regulators between the identified clusters was visualized and most of the regulators were upregulated in the C3 cluster (Fig. 2C). Subsequently, we performed a comprehensive assessment of the landscape of tumor infiltrating cells between the three clusters with the results revealing that the C3 and C2 clusters had higher abundance of different immune cells, while the C1 cluster only displayed endothelial cells as the most abundant cells. (Fig. 2D) We also performed differentially expressed gene analysis using whole-transcriptomic data. A clear pattern could be found by heatmap for differentially expressed genes at transcriptomic level (Additional file 2: Fig. S2B).

### The mutational pattern and immune landscape of the three identified clusters

Our results revealed that the three identified clusters displayed different enrichment mutations. In this regard, PBRM1 and PBRM were the top-ranked significant genes with higher mutation frequencies in the C1 and C3 clusters, respectively (Additional file 2: Figure S2A). Next, using the ESTIMATE method, we determined the abundance of immune and stromal cells present in the KIRC samples. As per the results, the C1 cluster was characterized by a higher stromal score, while the C3 cluster had a lower immune score (Additional file 2: Fig. S2B).

### Association between different identified clusters with clinicopathological characteristics, enriched pathways, and distinct gene signatures

**Fig. 3:**
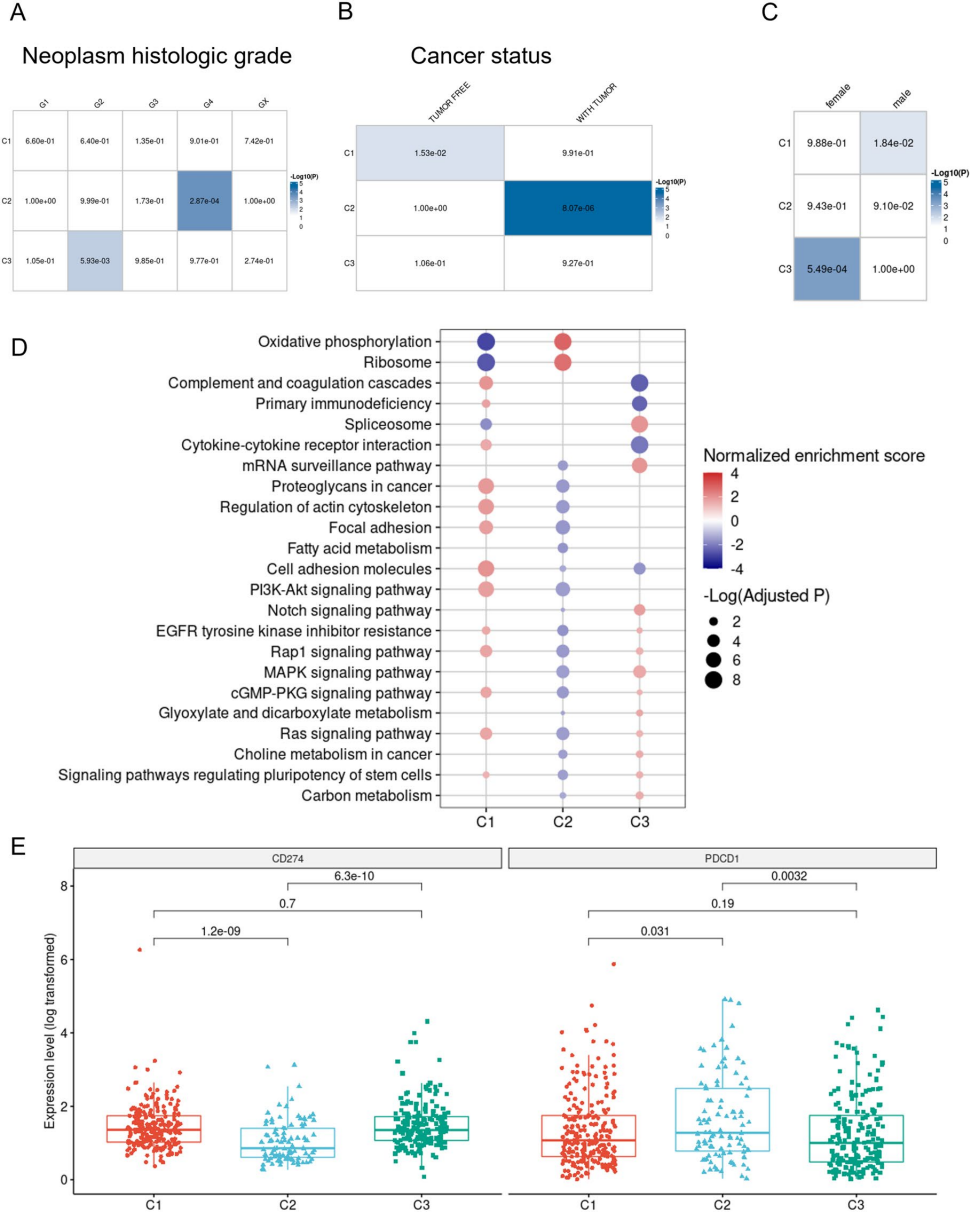
Clinicopathological characteristics and biological pathways of the three modification patterns. **A** Neoplasm histologic grades (G1, G2, G3, G4, GX) of three distinct clusters. **B** Cancer status of the different modification patterns (“Tumor free” and “With tumor” subgroups). **C** Gender of the three identified clusters. The significance of hypergeometric distribution test was shown in each cell. **D** Biological and KEGG pathway enrichment analysis of the three m1A modification patterns based on GSEA. **D** Immune checkpoint gene signature of the three identified clusters. *KEGG* Kyoto Encyclopedia of Genes and Genomes; *GSEA* Gene set enrichment analysis

Given the importance of m1A-related regulators in cancer progression, we subsequently assessed the relationship between modifications of the m1A-related regulatory genes and patient clinicopathological characteristics. The associations between m1A modification patterns and the clinicopathological features are illustrated in distinct heatmaps (p values of hypergeometric test were shown) in Fig. 3A–C. As shown in Fig. 3A–B**,** patients in the C2 cluster showed higher tumor grades, while the C1 cluster had more tumor-free patients across other clusters indicating the critical roles of m1A regulators in the progression of KIRC. In terms of gender, the C1 and C3 clusters were mostly comprised of males and females, respectively. Furthermore, functional enrichment analysis revealed that “oxidative phosphorylation”, “ribosome”, “complement and coagulation cascades”, “primary immunodeficiency”, “spliceosome”, “cytokine − cytokine receptor interaction”, and the “mRNA surveillance pathway” were among the top pathways, which were significantly different between the three clusters (Fig. 3D**)**. Finally, we identified the gene signatures of each of the three clusters. Our gene signature results showed that, in the C2 cluster, the expression level of PD-1 was higher, while its ligand, PDL-1, showed a lower expression, as compared to the other clusters.

### Subtype of m1A regulators was associated with KIRC drug resistance and prognosis

**Fig. 4:**
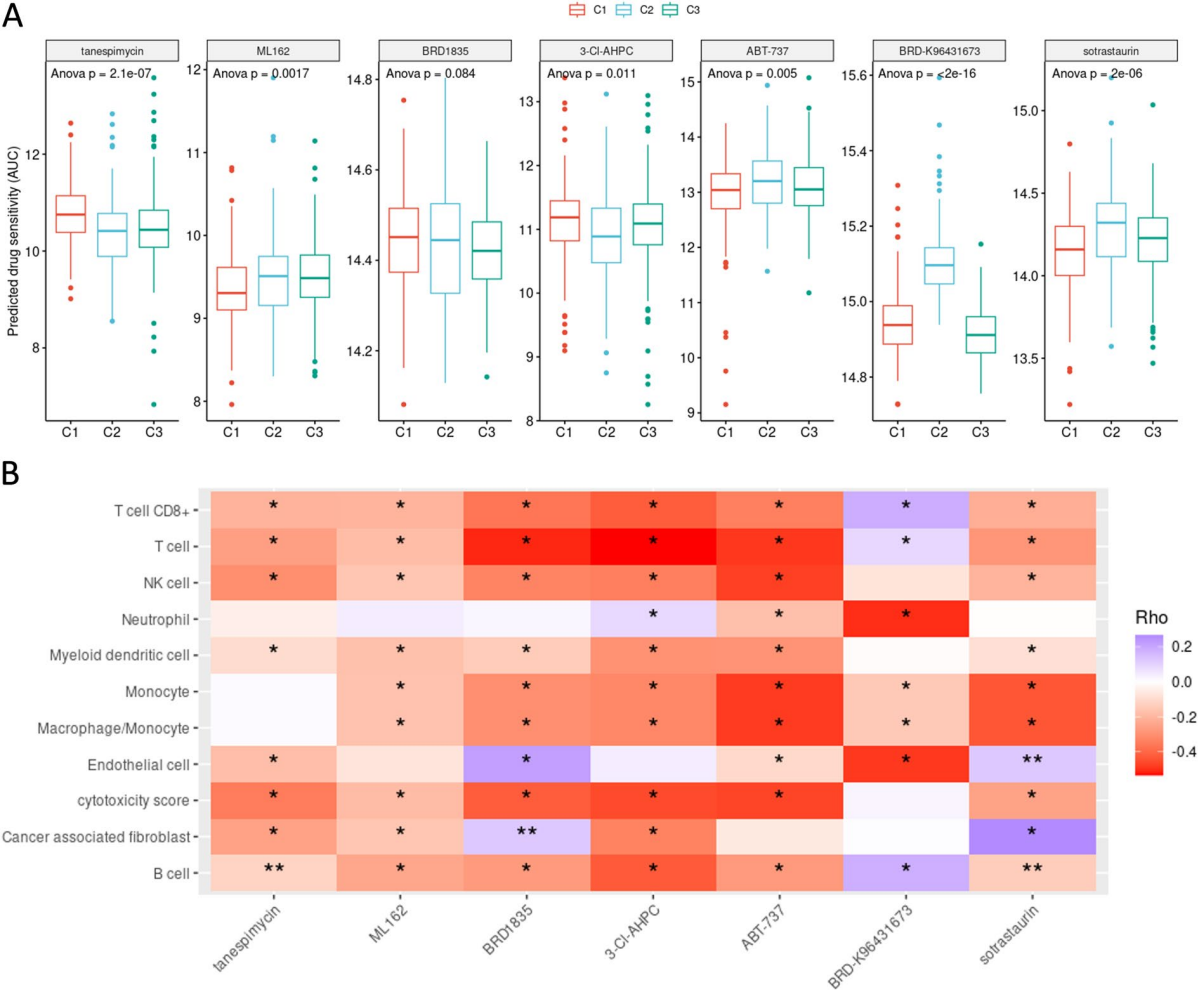
Correlation between immune cell infiltration and chemotherapy response; **A** Assessment of chemotherapy drug sensitivity was performed using Area Under the Curve (AUC) values derived from predictive modeling. The analysis was conducted for seven common chemotherapy drugs (Tanespimycin, ML162, BRD1835, 3-CIAHPC, ABT-737, BRD-K96431673, and Sotrastaurin), and patients were stratified based on their clustering profiles. The higher AUC values observed within specific clusters suggest a heightened resistance to certain chemotherapy drugs. **B** To explore the potential correlation between immune cell populations and drug response, Spearman's correlation analysis was conducted and represented as a heatmap. The heatmap illustrates the associations between immune cell infiltration and chemotherapy response, providing insights into the interplay between immune populations and drug efficacy. Notably, distinct immune cell types exhibited varying degrees of infiltration in response to different chemotherapy agents

Given our findings that the identified clusters exhibit different compositions of immune cells, we checked whether these differences correlate with response to treatment in these patients. Seven common chemotherapy drugs for which the required data was available were selected and the AUC values were calculated to predict sensitivity to each drug. Based on the results, patients within certain clusters show higher levels of resistance to certain chemotherapy drugs as indicated by their higher AUC values (Fig. 4A). To further investigate the possible correlation of different immune populations with response to these drugs, Spearman`s correlation test was performed, and the data was presented as a heatmap (Fig. 4B). Our analysis unveiled noteworthy associations, indicating that specific immune cell types exhibited varying degrees of infiltration following exposure to chemotherapy drugs. Particularly, endothelial cells and cancer-associated fibroblasts demonstrated increased infiltration subsequent to treatment with BRD-1835 and Sotrastaurin. On the other hand, CD8 + T-cells, T-cells, and B-cells displayed enhanced infiltration in response to the chemotherapy agent, BRD-k96431673. These findings offer valuable insights into the intricate interplay between chemotherapy agents and immune cell populations within the tumor microenvironment. The observed variations in immune cell infiltration may be indicative of drug-specific effects on the immune response, potentially contributing to the differential therapeutic outcomes of these agents. The heightened presence of endothelial cells and cancer-associated fibroblasts could be suggestive of their potential roles in the response to BRD-1835 and Sotrastaurin, possibly influencing the tumor's vascular microenvironment and fibrotic interactions. Conversely, the increased infiltration of CD8 + T-cells, T-cells, and B-cells following treatment with BRD-k96431673 might point towards an enhanced immunogenic response, implying the drug's impact on modulating the immune cell composition to facilitate a more robust antitumor immune activity.

**Fig. 5:**
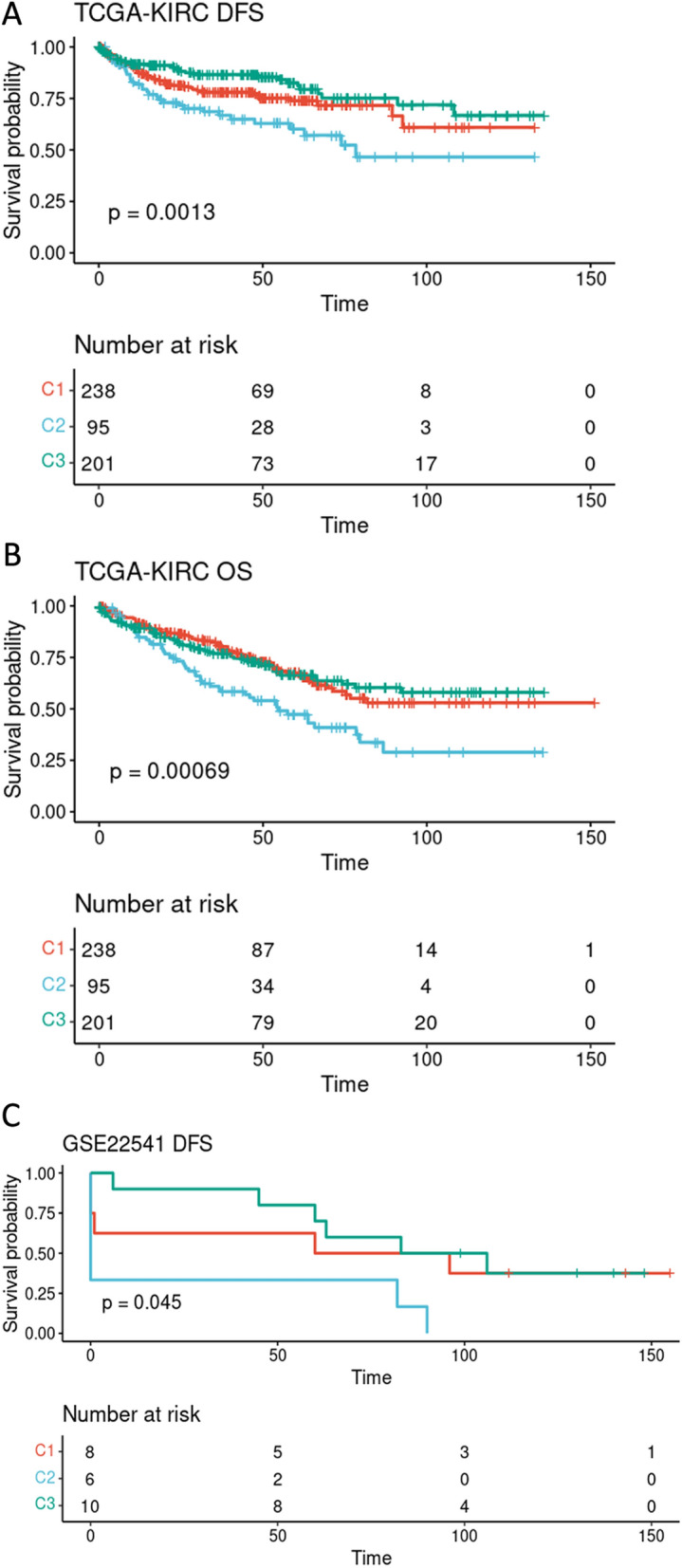
Association of the m1A expression pattern with disease free survival and overall survival. **A** Association of the three identified modification patterns and DFS in TCGA-KIRC cohorts. **B** Correlation of m1A expression pattern and OS in the TCGA-KIRC cohort. **C** Association of the m1A expression pattern and DFS in the GSE22541 patients. *DFS* Disease free survival, *OS* Overall survival

To assess the prognostic significance of the three distinct clusters in KIRC, we performed a univariate cox regression analysis on both the TCGA − KIRC and GSE22541 cohorts to identify survival-associated clusters. As shown in Fig. 5A, B, the clusters C3 and C2 in the TCGA-KIRC cohort showed better and worse DFS and overall survival (OS) rates, respectively. Similarly, among the GSE22541 patients, the C3 cluster had a better DFS rate, while the C2 cluster showed a poorer DFS as compared to the other clusters (Fig. 5C**)**. Taken together, these findings indicate the fact that the expression patterns of m1A regulators are associated with prognosis in KIRC patients.

## Discussion

RNA modification has been emerged as a hot topic of “RNA epitranscriptomics”, also known as “RNA epigenetics”, defined as different types of biochemical modifications in different coding and noncoding RNAs [27]. Generation of a N1-methyladenosine, the so-called m1A methylation, is among the post-transcriptional RNA modifications and has been shown to play an essential role in cancer progression [28]. As a new study focus, m1A RNA methylation has been attributed to a number of key biological processes, including protein translation [29] and RNA metabolism [30]. Nevertheless, investigations on RNA modifications in KIRC patients have mostly focused on a few RNA modification regulators, particularly m6A-related regulators, with the functions of other types of RNA modifications and their interactions remaining unexplored.

In the present study, we aimed to analyze the alterations in m1A-related regulators in KIRC and its relationships with clinicopathological characteristics, using the TCGA-KIRC and GSE22541 databases. The differential expression, crosstalk, and potential prognostic values of the ten m1A-related regulatory genes in KIRC patients, as well as the associated biological activities and signaling pathways, were investigated. Ultimately, certain m1A modification patterns among KIRC patients were identified and through this classification, immune cell infiltration, gene signature, clinicopathological features, drug resistance and prognostic values of each cluster was investigated.

We first evaluated RNA expression of the ten m1A-related regulators in the TCGA-KIRC cohort and identified that 5 out of the 10 studied genes including TRMT10C, TRMT6, TRMT61B, YTHDF2, and YTHDF3 were up-regulated in these patients, compared to the healthy tissues. Unlike previous genes, which are categorized as readers and writers, both eraser genes (ALKBH1 and ALKBH3) were shown to be downregulated in KIRC patients. Demethylation by ALKBH1 has been shown to enhance the stability of tRNAs leading to decreased tRNA usage in the translation process [31]. In addition, individuals with pancreatic adenocarcinoma (PAAD), whose expression of the ‘‘eraser’’ gene ALKBH1 is low, have a worse prognosis in comparison to high expressers [24]. Furthermore, recent evidence has shown that demethylation by ALKBH3 may increase translation performance. Therefore, suppressing ALKBH3 may inhibit protein synthesis by increasing the quantity of m1A in tRNAs [15]. Moreover, mutation frequency analysis revealed that the mutation burden varied from 14 to 43% in 7 samples of the analyzed cohorts, and YTHDC1 had the highest mutation burden among the other m1A-related regulators. Zheng et al. also reported that the clinical stage of patients with pancreatic cancer is correlated with genetic variants in the m1A regulators, and copy number variations (CNVs) are strongly correlated with the expression of m1A regulators [24]. Li et al. conducted a thorough analysis of the correlation between the clinical information from 33 different cancer types from the TCGA and the molecular alterations of m1A-reated regulators. Their team discovered that m1A-related regulatory gene expression was associated with a number of carcinogenic pathways and patient OS, suggesting that m1A regulators may be used to predict prognosis in a variety of cancers and may possibly give rise to novel therapeutic targets [32]. In our analysis, we identified that TRMT6 and TRMT61A were associated with a poor prognosis in TCGA-KIRC patients based on the univariate cox regression analysis. Similarly, Wang et al. demonstrated that the deregulation of m1A regulators was significantly correlated with glioma tumorigenesis and progression. Moreover, their findings indicated that TRMT6 may be a potent biomarker for glioma prognosis [33]. In another parallel study, Wang et al. confirmed that TRMT6 and TRMT61A are overexpressed in advanced hepatocellular carcinoma (HCC) tumors and correlate adversely with HCC patient survival. In brief, they confirmed that TRMT6/TRMT61A increases m1A methylation in a subset of tRNAs to enhance PPAR translation, which in turn induces cholesterol production to activate Hedgehog signaling pathway, ultimately promoting self-renewal of liver cancer stem cells and HCC tumorigenesis [34]. Moreover, the relationship between m1A regulators and TME-infiltrating immune cells has remained poorly understood. We discovered a substantial correlation between the ten m1A regulators and the TME-infiltrating immune cells using the GSEA algorithm, suggesting the possible function of m1A regulators in the immunological TME and eventual progression of KIRC.

Here, we also identified three m1A modification patterns on the basis of the ten studied m1A regulators in the TCGA-KIRC patients, which we labeled as Cluster “C1”, Cluster “C2”, and Cluster “C3”. Growing evidence suggests that m1A modification patterns may impact different clinicopathological features including the infiltration of immune cells, tumor grade, cancer status, and prognosis of the patients [35]; consequently, the associations between RNA modification imposed by m1A methylation and the such characteristics has been emerging as a hot topic in cancer field. In this context, Liu et al. discovered three distinct m1A alteration patterns in ovarian cancer that may forecast patient survival, tumor grade, and stage [36]. Within the distinguished clusters, patients in the C2 cluster displayed the poorest prognosis and the highest tumor grades among other clusters in the KIRC cohorts. Meanwhile, C1 showed a higher stromal score, while Cluster C3 had lower immune score based on the ESTIMATE algorithm. Regarding the poor prognosis of the C2 cluster, TME immune cell infiltration data revealed that this cluster has the highest abundance of B lymphocytes and neutrophils. Tumor-suppressive effects of B lymphocytes are highlighted by a number of studies, which have established a correlation of B lymphocytes with longer survival rates in multiple malignancies [37–39]; however, in other tumors, the relationship between B lymphocytes and prognosis was less evident, and some investigations have even linked B lymphocytes to a worse prognosis [40]. Interestingly, as an indicator of inflammation, tumor-infiltrating neutrophils (TINs) have been correlated with poor prognosis in different types of malignancies. With regard to KIRC, Tessier-Cloutier et al. reported that TINs can act as independent indicators of poor prognosis, which can be clinically utilized in the biopsy or fine-needle aspiration [41]. Paradoxically, based on the ESTIMATE algorithm, C2 had the highest immune score in comparison to C1 and C3 clusters. As previously reported, the presence of numerous immune cells preserved in the stroma around tumor cell nests may activate the stroma of TME, hence hindering an effective immune response [42]. Consequently, we hypothesized that the activation of stromal cells in the C2 cluster decreased the antitumor activity of immune cells. Furthermore, GSEA analysis demonstrated that the three m1A modification patterns followed statistically distinct pathways. C3 with the best OS included spliceosome and mRNA surveillance pathways, C1 with the poorer prognosis included proteoglycan in cancer, actin cytoskeleton control, and cell adhesion molecules, and C2 with the worst prognosis was mainly enriched in oxidative phosphorylation and ribosome pathways.

Our comprehensive analysis revealed significant findings through functional enrichment assessment, with prominent involvement of key pathways such as ‘‘oxidative phosphorylation’’ and ‘‘complement and coagulation cascades.’’ These results align notably with the underlying nature of RCC, which fundamentally manifests as a metabolic disorder marked by the strategic reprogramming of energetic metabolism [43]. Of note, this metabolic reprogramming equips tumor cells with the means to endure conditions of energy scarcity and hypoxia, foster the synthesis of essential biomolecular (such as proteins, DNA, and membranes) to facilitate rapid proliferation, and elude host immunosurveillance while concurrently mitigating oxidative stress [44]. A consequential aspect of this metabolic shift is an alteration in levels of various biochemical entities, including enzymes, substrates, metabolic intermediates, and ultimate products. These altered metabolomic profiles offer compelling prospects as diagnostic biomarkers, enabling refined assessments of tumor behavior encompassing aggressiveness, prognostic markers, and responsiveness to therapeutic interventions.

In a study conducted by Torsello et al., it was demonstrated that high glucose levels induce notable alterations in mesenchymal markers, including Snail1, miRNA210, and Vimentin, within primary tubular cells. This coincides with reduced N-cadherin expression and migration capabilities, coupled with altered inflammatory cytokine secretion, effectively modulating the proliferation and migration of fibroblasts. This mechanism evokes an activated state of partial epithelial − mesenchymal transition (EMT), a process implicated in the pathogenesis of diabetic nephropathy [45]. The work of Bianchi et al. corroborates the grade-dependent prominence of the Warburg effect and fatty acid oxidation in ccRCC [46].

Furthermore, Ragone et al. observed notable increases in levels of creatine, alanine, lactate, and pyruvate, coupled with decreases in hippurate, citrate, and betaine across all patients diagnosed with ccRCC. Within their study, an intricate network analysis established connections between the majority of these metabolites and critical aspects, such as glomerular injury, renal inflammation, and renal necrosis/cell death. Notably, through an intersection of metabolomic data with transcriptomic information extracted from CD133 + /CD24 + tumoral renal stem cells, isolated from ccRCC patients, the study identified a shared subset of genes and metabolites that showcased distinct regulatory patterns in ccRCC cases. These regulations were found to be associated with significant pathways, including HIF-α signaling, methionine and choline degradation, and acetyl-CoA biosynthesis [47].

By exploring glycolytic enzyme levels, Lucarelli et al. associate elevated levels with reduced survival outcomes in ccRCC patients, signifying the influence of oncogenic signaling pathways in driving the glucose metabolism shift [48]. Another study by Lucarelli et al. further unveiled an amplified glucose uptake and utilization signature in ccRCC, accompanied by perturbations in the pentose phosphate pathway. The central role of NADH dehydrogenase (ubiquinone) 1 alpha subcomplex 4-like 2 (NDUFA4L2) is highlighted in sustaining angiogenesis, chemoresistance, and mitochondrial dysfunction. Notably, inhibiting NDUFA4L2 engenders changes in cell viability, mitochondrial mass, and ROS generation under hypoxic conditions [49].

Regarding lipid metabolism, Bombelli et al. reported that ccRCC exhibits a distinctive gene expression profile indicative of adipogenesis. Notably, their investigation highlighted the down-regulation of the phospholipid-binding protein annexin A3 (AnxA3), known for its role as a negative regulator of adipocyte differentiation, in RCC. Intriguingly, the study revealed a differential expression pattern for two isoforms of AnxA3, measuring 36 and 33 kDa, respectively. Moreover, they observed a correlation between the increased accumulation of lipids within ccRCC cells and a reduction in the 36/33 isoform ratio of AnxA3. These observations collectively suggest that the 36 kDa isoform of AnxA3 might exert a negative influence on the response to adipogenic treatment, implying its potential role as a regulator that curtails lipid storage in ccRCC cells [50].

In addition, renal cell carcinoma is one of the most immune-infiltrated tumors. Our findings are also well in line with the hot nature of RCC tumor that generally show high levels of immune infiltration. Consistent with this viewpoint, a study conducted by Zhang et al. showed a significant correlation between the Holliday junction recognition protein (HJURP) and the progression of tumors across various malignancies, frequently indicating a poor prognosis [51]. This association extends to the infiltration of diverse immune cells and the expression of a broad array of genetic markers associated with immune cells. In addition, Tamma et al. contributed insights into microvascular density, macrophage presence, and mast cell involvement in human ccRCC both with and without bevacizumab treatment. Their findings point to the potential mechanisms underlying bevacizumab's antiangiogenic impact, encompassing its direct influence on tumor cell-derived angiogenic cytokines and its indirect effect on pro-angiogenic factor release by inflammatory stromal cells [52]. Notably, Netti et al. unveiled the role of PTX3, an innate immune regulator from the pentraxin family, in ccRCC's microenvironment. This protein disrupts immunoflogosis by triggering the activation of the classical pathway of the complement system (C1q), leading to the subsequent release of pro-angiogenic factors (C3a, C5a) [53].

Interestingly, an intricate interplay emerges between immune infiltration levels and prognosis. The cluster exhibiting the most adverse prognosis (C2) paradoxically showcased the lowest immune infiltration, a phenomenon coupled with dysregulation in specific metabolic pathways like oxidative phosphorylation and ribosome pathways. In parallel, Lucarelli et al. identified a nearly sixfold elevation of Kynurenine in ccRCC, coupled with diminished levels of tryptophan. Notably, Kynurenine exerts pivotal influence over RC survival, migration, and chemoresistance, mediated through interactions with the aryl hydrocarbon receptor. Moreover, the Kynurenine-to-tryptophan ratio has emerged as a promising indicator for assessing the aggressiveness of ccRCC, thereby functioning as a prognostic factor for both cancer-specific and progression-free survival [54].

Undoubtedly, it is crucial to emphasize the significant role played by the tumor microenvironment in shaping the biological behavior of the disease, potentially affecting responses to systemic therapeutic interventions. Expanding upon this perspective, the work of Lucarelli et al. sheds additional light on the capacity of MUC1 to govern the immunoflogosis within the microenvironment of ccRCC. This protein operates by activating the classical pathway of the complement system; thereby, intricately influencing the composition of the immune infiltrate and promoting an environment characterized by immunological suppression [55].

In summation, our findings seamlessly align with the narrative of RCC as an immune-rich tumor type, characterized by complex interactions between immune cells, metabolic pathways, and the tumor microenvironment. This intricate interplay not only shapes disease progression, but also holds the potential to guide the development of targeted therapeutic strategies.

While our study provides valuable insights into the role of m1A RNA modification patterns in KIRC, there are certain limitations that should be acknowledged. First, our study is based on the retrospective data analysis, which might introduce biases and confounding factors inherent to this type of research. Moreover, the interplay between m1A regulators and other molecular pathways in the complex tumor microenvironment warrants deeper investigation. In addition, our study focused on a specific set of m1A-related regulatory genes; exploring a broader set of regulators and their interactions could provide a more comprehensive understanding. Furthermore, the immune landscape and tumor microenvironment are highly dynamic and subject to variation over time, which might affect the stability of identified patterns and correlations. Last but not least, while our findings provide potential avenues for prognostic prediction and personalized treatment strategies, validation on larger and independent cohorts is essential for robust clinical translation.

## Conclusion

In summary, the present research identifies m1A regulators in KIRC across numerous aspects and substantiates their significance in determining prognosis and immune performance. To our knowledge, the present work is the first to report the complex functions and wide-ranging interconnections of ten different types of m1A-related RNA modifications in KIRC. We identified three different RNA modification patterns, their underlying biological pathways, their correlations with clinicopathological features, and their potential prognostic values in the KIRC patients. This work emphasizes the importance of ten different RNA modifications in KIRC and provides a novel insight for future research, herein.

### Supplementary Information

**Additional file 1: Figure S1.** Mutations in m1a regulators (seven samples have mutation in five m1A regulators).

**Additional file 2: Figure S2.** **A** Barplot shows the mutation frequency of genes which were significantly different between subtypes. **B** Boxplots show the stromal, immune and ESTIMATE score were significantly differetny across three subtypes. **C** Heatmap shows the expression pattern of differentially expressed  genes across three subtypes at transcriptomic level.

## Data Availability

The datasets or codes used and/or analyzed during the current study are available from the corresponding author on reasonable request.

## References

[1] Turajlic S, Swanton C, Boshoff C (2018). Kidney cancer: the next decade. J Exp Med.

[2] Pallagani L, Choudhary GR, Himanshu P, Madduri VK, Singh M, Gupta P (2021). Epidemiology and clinicopathological profile of renal cell carcinoma: a review from tertiary care referral centre. J Kidney Cancer VHL.

[3] Tacconi EM, Tuthill M, Protheroe A (2020). Review of adjuvant therapies in renal cell carcinoma: evidence to date. Onco Targets Ther.

[4] Lotan Y, Karam JA, Shariat SF, Gupta A, Roupret M, Bensalah K (2016). Renal-cell carcinoma risk estimates based on participants in the prostate lung colorectal, and ovarian cancer screening trial and national lung screening trial urologic oncology seminars and original investigations.

[5] Ricketts CJ, Linehan WM (2015). Gender specific mutation incidence and survival associations in clear cell renal cell carcinoma (CCRCC). PLoS ONE.

[6] Yin L, Li W, Wang G, Shi H, Wang K, Yang H (2019). NR1B2 suppress kidney renal clear cell carcinoma (KIRC) progression by regulation of LATS 1/2-YAP signaling. J Exp Clin Cancer Res.

[7] Hsieh JJ, Purdue MP, Signoretti S, Swanton C, Albiges L, Schmidinger M (2017). Renal cell carcinoma. Nat Rev Dis Primers.

[8] Rosenblum D, Peer D (2014). Omics-based nanomedicine: the future of personalized oncology. Cancer Lett.

[9] Frantzi M, Hupe MC, Merseburger AS, Schanstra JP, Mischak H, Latosinska A (2020). Omics derived biomarkers and novel drug targets for improved intervention in advanced prostate cancer. Diagnostics.

[10] Fujiwara N, Qian T, Koneru B, Hoshida Y (2020). Omics-derived hepatocellular carcinoma risk biomarkers for precision care of chronic liver diseases. Hepatol Res.

[11] Qu Y, Feng J, Wu X, Bai L, Xu W, Zhu L (2022). A proteogenomic analysis of clear cell renal cell carcinoma in a Chinese population. Nat Commun.

[12] Ferro M, Crocetto F, Barone B, Del Giudice F, Maggi M, Lucarelli G (2023). Artificial intelligence and radiomics in evaluation of kidney lesions: a comprehensive literature review. Ther Adv Urol.

[13] Nachtergaele S, He C (2017). The emerging biology of RNA post-transcriptional modifications. RNA Biol.

[14] Roundtree IA, Evans ME, Pan T, He C (2017). Dynamic RNA modifications in gene expression regulation. Cell.

[15] Zhang C, Jia G (2018). Reversible RNA modification N1-methyladenosine (m1A) in mRNA and tRNA. Genom Proteom Bioinform.

[16] Wang S, Sun C, Li J, Zhang E, Ma Z, Xu W (2017). Roles of RNA methylation by means of N6-methyladenosine (m6A) in human cancers. Cancer Lett.

[17] Zhao BS, He C (2015). Pseudouridine in a new era of RNA modifications. Cell Res.

[18] Trixl L, Lusser A (2019). The dynamic RNA modification 5-methylcytosine and its emerging role as an epitranscriptomic mark. Wiley Interdiscipl Rev RNA.

[19] Xie S, Chen W, Chen K, Chang Y, Yang F, Lin A (2020). Emerging roles of RNA methylation in gastrointestinal cancers. Cancer Cell Int.

[20] Dominissini D, Rechavi G (2017). Loud and clear epitranscriptomic m1A signals: now in single-base resolution. Mol Cell.

[21] Song J, Yi C (2017). Chemical modifications to RNA: a new layer of gene expression regulation. ACS Chem Biol.

[22] Bao G, Li T, Guan X, Yao Y, Liang J, Xiang Y (2022). Comprehensive analysis of immune profiles and clinical significance of m1A regulators in lung adenocarcinoma. Frontiers Oncol.

[23] Shi Q, Xue C, Yuan X, He Y, Yu Z (2020). Gene signatures and prognostic values of m1A-related regulatory genes in hepatocellular carcinoma. Sci Rep.

[24] Zheng Q, Yu X, Zhang Q, He Y, Guo W (2021). Biosci Rep.

[25] Gao L, Chen R, Sugimoto M, Mizuta M, Kishimoto Y, Omori K (2021). The Impact of m1A methylation modification patterns on tumor immune microenvironment and prognosis in oral squamous cell carcinoma. Int J Mol Sci.

[26] Su G, Liu T, Han X, Sun H, Che W, Hu K (2021). YTHDF2 is a potential biomarker and associated with immune infiltration in kidney renal clear cell carcinoma. Frontiers Pharmacol.

[27] Barbieri I, Kouzarides T (2020). Role of RNA modifications in cancer. Nat Rev Cancer.

[28] Xiong X, Li X, Yi C (2018). N1-methyladenosine methylome in messenger RNA and non-coding RNA. Curr Opin Chem Biol.

[29] Song D, Shyh-Chang N (2022). An RNA methylation code to regulate protein translation and cell fate. Cell Prolifer.

[30] Chatterjee B, Shen C-KJ, Majumder P (2021). RNA modifications and RNA metabolism in neurological disease pathogenesis. Int J Mol sci.

[31] Li Y, Zheng D, Wang F, Xu Y, Yu H, Zhang H (2019). Expression of demethylase genes, FTO and ALKBH1, is associated with prognosis of gastric cancer. Dig Dis Sci.

[32] Li J, Zhang C, Yuan X, Cao Y (2021). Molecular characteristics of N1-methyladenosine regulators and their correlation with overall cancer survival. DNA Cell Biol.

[33] Wang B, Niu L, Wang Z, Zhao Z (2021). RNA m1A methyltransferase TRMT6 predicts poorer prognosis and promotes malignant behavior in glioma. Frontiers Mol Biosci.

[34] Wang Y, Wang J, Li X, Xiong X, Wang J, Zhou Z (2021). N1-methyladenosine methylation in tRNA drives liver tumourigenesis by regulating cholesterol metabolism. Nat Commun.

[35] Miao Y-D, Mu L-J, Tang X-L, Wang J-T, Wu J-J, Chen Y-G, et al. 2021. N1-Methyladenosine (m1A) regulator-mediated methylation modification modes and tumor microenvironment infiltration characteristics in head and neck squamous cell Carcinomas. 2021.

[36] Liu J, Chen C, Wang Y, Wei J, Bai J (2021). Comprehensive of N1-methyladenosine modifications patterns and immunological characteristics in ovarian cancer. Frontiers Immunol.

[37] Ni Z, Xing D, Zhang T, Ding N, Xiang D, Zhao Z (2021). Tumor-infiltrating B cell is associated with the control of progression of gastric cancer. Immunol Res.

[38] Valiev I, Kotlov N, Belozerova A, Lopareva A, Butusova A, Samarina N (2022). B cell content in the tumor microenvironment is associated with improved survival in stage II lung adenocarcinoma. Cancer Res.

[39] Qin Y, Peng F, Ai L, Mu S, Li Y, Yang C (2021). Tumor-infiltrating B cells as a favorable prognostic biomarker in breast cancer: a systematic review and meta-analysis. Cancer Cell Int.

[40] Sjöberg E, Frödin M, Lövrot J, Mezheyeuski A, Johansson M, Harmenberg U (2018). A minority-group of renal cell cancer patients with high infiltration of CD20+ B-cells is associated with poor prognosis. Br J Cancer.

[41] Tessier-Cloutier B, Twa DD, Marzban M, Kalina J, Chun HJE, Pavey N (2021). The presence of tumour-infiltrating neutrophils is an independent adverse prognostic feature in clear cell renal cell carcinoma. J Pathol Clin Res.

[42] Chen DS, Mellman I (2017). Elements of cancer immunity and the cancer–immune set point. Nature.

[43] Zaravinos A, Deltas C (2014). ccRCC is fundamentally a metabolic disorder. Cell Cycle.

[44] Miranda-Galvis M, Teng Y (2020). Targeting hypoxia-driven metabolic reprogramming to constrain tumor progression and metastasis. Int J Mol Sci.

[45] Torsello B, De Marco S, Bombelli S, Cifola I, Morabito I, Invernizzi L (2023). High glucose induces an activated state of partial epithelial-mesenchymal transition in human primary tubular cell cultures. PLoS ONE.

[46] Bianchi C, Meregalli C, Bombelli S, Di Stefano V, Salerno F, Torsello B (2017). The glucose and lipid metabolism reprogramming is grade-dependent in clear cell renal cell carcinoma primary cultures and is targetable to modulate cell viability and proliferation. Oncotarget.

[47] Ragone R, Sallustio F, Piccinonna S, Rutigliano M, Vanessa G, Palazzo S (2016). Renal cell carcinoma: a study through NMR-based metabolomics combined with transcriptomics. Diseases.

[48] Lucarelli G, Galleggiante V, Rutigliano M, Sanguedolce F, Cagiano S, Bufo P (2015). Metabolomic profile of glycolysis and the pentose phosphate pathway identifies the central role of glucose-6-phosphate dehydrogenase in clear cell-renal cell carcinoma. Oncotarget.

[49] Lucarelli G, Rutigliano M, Sallustio F, Ribatti D, Giglio A, Signorile ML (2018). Integrated multi-omics characterization reveals a distinctive metabolic signature and the role of NDUFA4L2 in promoting angiogenesis, chemoresistance, and mitochondrial dysfunction in clear cell renal cell carcinoma. Aging.

[50] Bombelli S, Torsello B, De Marco S, Lucarelli G, Cifola I, Grasselli C (2020). 36-kDa annexin A3 isoform negatively modulates lipid storage in clear cell renal cell carcinoma cells. Am J Pathol.

[51] Yuan JS, Chen ZS, Wang K, Zhang ZL (2020). Holliday junction-recognition protein modulates apoptosis, cell cycle arrest and reactive oxygen species stress in human renal cell carcinoma. Oncol Rep.

[52] Tamma R, Rutigliano M, Lucarelli G, Annese T, Ruggieri S, Cascardi E (2019). Microvascular density, macrophages, and mast cells in human clear cell renal carcinoma with and without bevacizumab treatment urologic oncology seminars and original investigations.

[53] Netti GS, Lucarelli G, Spadaccino F, Castellano G, Gigante M, Divella C (2020). PTX3 modulates the immunoflogosis in tumor microenvironment and is a prognostic factor for patients with clear cell renal cell carcinoma. Aging.

[54] Lucarelli G, Rutigliano M, Ferro M, Giglio A, Intini A, Triggiano F (2017). Activation of the kynurenine pathway predicts poor outcome in patients with clear cell renal cell carcinoma urologic oncology seminars and original investigations.

[55] Lucarelli G, Rutigliano M, Loizzo D, di Meo NA, Lasorsa F, Mastropasqua M (2022). MUC1 tissue expression and its soluble form CA15-3 identify a clear cell renal cell carcinoma with distinct metabolic profile and poor clinical outcome. Int J Mol Sci.

