# Supplementary figures and images for: m1A Regulatory gene signatures are associated with certain immune cell compositions of the tumor microenvironment and predict survival in kidney renal clear cell carcinoma
Source: Eur J Med Res. 2023 Sep 7;28:321. doi: 10.1186/s40001-023-01292-3 (PMC10483733; doi:10.1186/s40001-023-01292-3)

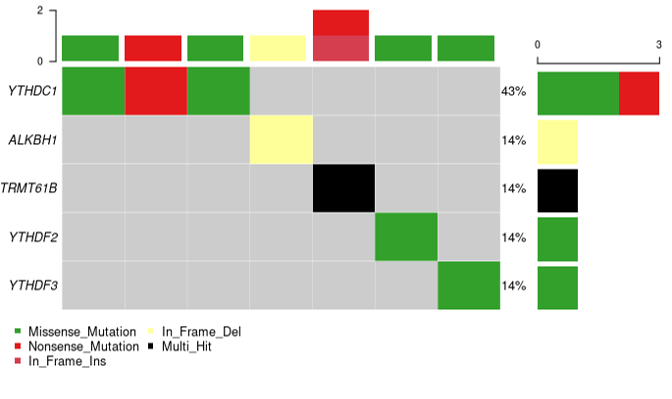

Supplement: Supplementary file 1 — Additional file 1: Figure S1. Mutations in m1a regulators (seven samples have mutation in five m1A regulators). [file 40001_2023_1292_MOESM1_ESM.tif]

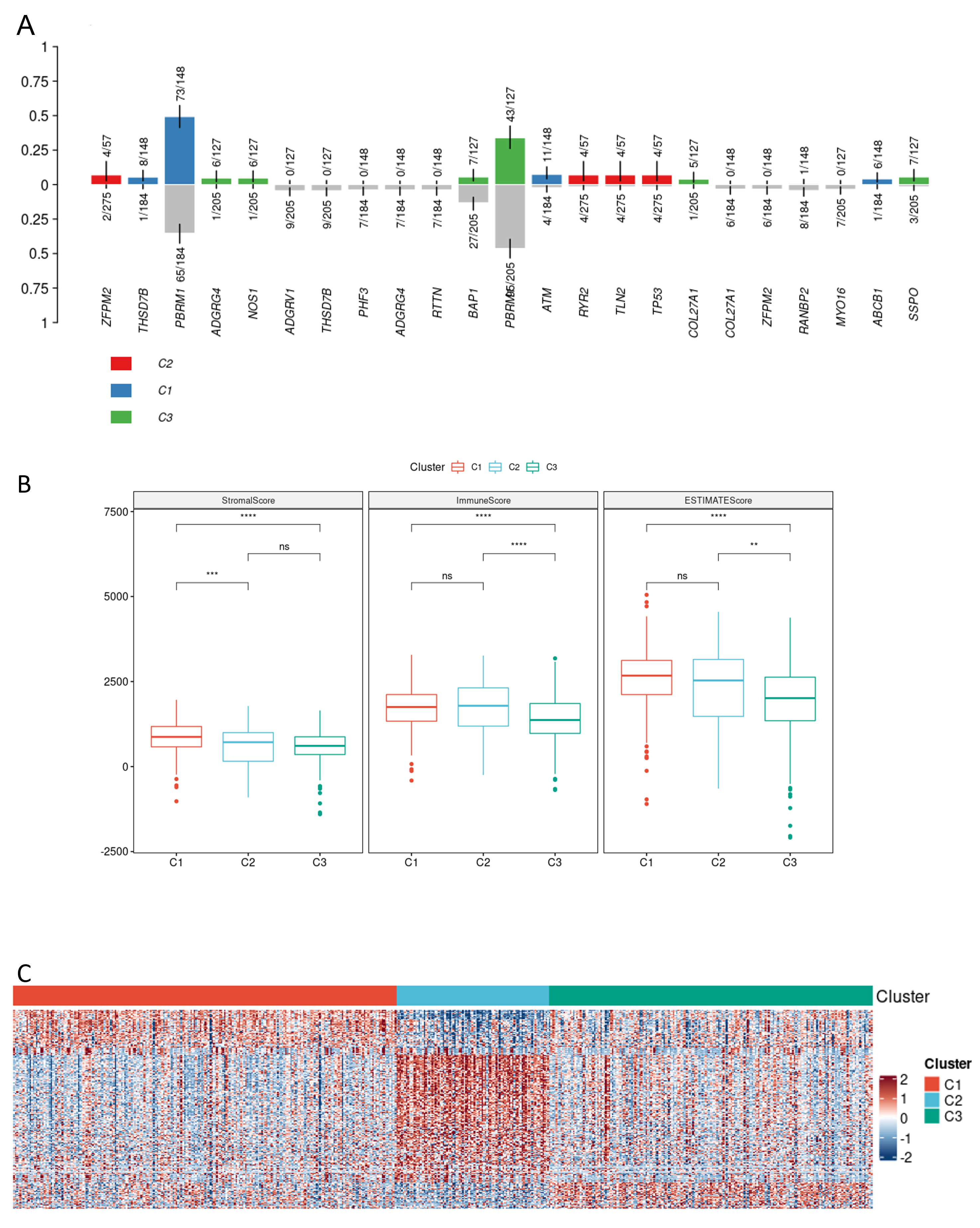

Supplement: Supplementary file 2 — Additional file 2: Figure S2. A Barplot shows the mutation frequency of genes which were significantly different between subtypes. B Boxplots show the stromal, immune and ESTIMATE score were significantly differetny across three subtypes. C Heatmap shows the expression pattern of differentially expressed genes across three subtypes at transcriptomic level. [file 40001_2023_1292_MOESM2_ESM.tif]
